# Changes in Biomarkers and Hemodynamics According to Antibiotic Susceptibility in a Model of Bacteremia

**DOI:** 10.1128/spectrum.00864-22

**Published:** 2022-07-11

**Authors:** Inwon Park, Dongsung Kim, Jae Hyuk Lee, Sung Jin Park, Hwain Jeong, Sumin Baek, Seonghye Kim, Serin Kim, Ji Eun Hwang, Hyuksool Kwon, Joo H. Kang, You Hwan Jo

**Affiliations:** a Department of Emergency Medicine, Seoul National University Bundang Hospital, Gyeonggi-do, Republic of Korea; b Department of Emergency Medicine, Seoul National University College of Medicine, Seoul, Republic of Korea; c Department of Biomedical Engineering, Ulsan National Institute of Science and Technology (UNIST), Ulsan, Republic of Korea; Louis Stokes Cleveland VAMC

**Keywords:** antibacterial agents, biomarkers, drug resistance, bacterial, sepsis, triggering receptor expressed on myeloid cells-1

## Abstract

Proper selection of susceptible antibiotics in drug-resistant bacteria is critical to treat bloodstream infection. Although biomarkers that guide antibiotic therapy have been extensively evaluated, little is known about host biomarkers targeting *in vivo* antibiotic susceptibility. Therefore, we aimed to evaluate the trends of hemodynamics and biomarkers in a porcine bacteremia model treated with insusceptible antibiotics compared to those in susceptible models. Extended-spectrum β-lactamase (ESBL)-producing Escherichia coli (E. coli, 5.0 * 10^9 CFU) was intravenously administered to 11 male pigs. One hour after bacterial infusion, pigs were assigned to two groups of antibiotics, ceftriaxone (*n* = 6) or ertapenem (*n* = 5). Pigs were monitored up to 7 h after bacterial injection with fluid and vasopressor support to maintain the mean arterial blood pressure over 65 mmHg. Blood sampling for blood culture and plasma acquisition was performed before and every predefined hour after E. coli injection. Cytokine (tumor necrosis factor-α, interleukin [IL]-1β, IL-6, IL-8, IL-10, C-reactive protein, procalcitonin, presepsin, heparan sulfate, syndecan, and soluble triggering receptor expressed on myeloid cells-1 [sTREM-1]) levels in plasma were analyzed using enzyme-linked immunosorbent assays. Bacteremia developed after intravenous injection of E. coli, and negative conversion was confirmed only in the ertapenem group. While trends of other biomarkers failed to show differences, the trend of sTREM-1 was significantly different between the two groups (*P = *0.0001, two-way repeated measures analysis of variance). Among hemodynamics and biomarkers, the sTREM-1 level at post 2 h after antibiotics administration represented a significant difference depending on susceptibility, which can be suggested as a biomarker candidate of *in vivo* antibiotics susceptibility. Further clinical studies are warranted for validation.

**IMPORTANCE** Early and appropriate antibiotic treatment is a keystone in treating patients with sepsis. Despite its importance, blood culture which requires a few days remains as a pillar of diagnostic method for microorganisms and their antibiotic susceptibility. Whether changes in biomarkers and hemodynamics indicate treatment response of susceptible antibiotic compared to resistant one is not well understood to date. In this study using extended-spectrum β-lactamase -producing E. coli bacteremia porcine model, we have demonstrated the comprehensive cardiovascular hemodynamics and trends of plasma biomarkers in sepsis and compared them between two groups with susceptible and resistant antibiotics. While other hemodynamics and biomarkers have failed to differ, we have identified that levels of soluble triggering receptor expressed on myeloid cells-1 (sTREM-1) significantly differed between the two groups over time. Based on the data in this study, trends of sTREM-1 obtained before the antibiotics and 2~4 h after the antibiotics could be a novel host biomarker that triggers the step-up choice of antibiotics.

## INTRODUCTION

Early administration of adequate antimicrobials, ideally within 1 h of sepsis recognition, is one of the most efficient interventions to reduce mortality in patients with sepsis (1–3). In the empirical phase of sepsis, when the causative pathogen and its susceptibilities are unknown, the optimal selection of antibiotics generally depends on the local prevalence of resistant pathogens, host risk factors for resistant organisms, and the severity of sepsis (4–6). Blood culture remains the current gold standard diagnostic method for reporting types of microorganisms and antibiotic susceptibility. However, the method requires considerable time for clinicians to adjust antibiotic selection (7, 8). In addition, blood culture appears negative in a substantial proportion of patients with sepsis, resulting in the decision of antibiotic step-up being solely dependent on either the clinical deterioration or the experience of the clinician (9, 10). Therefore, along with the development of state-of-the-art technology for shortening the detection time and increasing the accuracy for revealing microorganisms, novel indicators should be explored based on the response of the host to sepsis (7, 11, 12).

Whether changes in hemodynamics and biomarkers could be indicative of antibiotic susceptibility and further reduction of bacterial load in sepsis is unknown (12). Direct comparisons, including longitudinal analyses of hemodynamics and biomarkers according to susceptible antibiotics in bacteremia, are lacking in both clinical and preclinical studies (13, 14). Investigation of their dynamics over the progression of sepsis would facilitate the understanding of the pathophysiology and impact of susceptible antibiotics (13). Furthermore, if treatment response variables of the host could be characterized, it might aid clinical decisions with respect to both proper selection of antibiotics and avoidance of unnecessary antibiotic use (15).

However, most treatment response biomarkers are based on daily measurements, and benefits may be limited in patients with sepsis representing multiorgan failures and death within a day (16, 17). Hemodynamic variables according to the susceptibility of antibiotics were challenging to evaluate due to the presence of many confounding factors in the clinical field (18). Evidence is lacking in regard to the full dynamics of either biomarkers or hemodynamics in patients with sepsis with respect to *in vivo* antibiotic susceptibility. Thus, to understand the changes in hemodynamics and biomarkers after antibiotic treatment that can be further developed as an optimization of antibiotic selection in patients with sepsis, the differentiation of hemodynamic characteristics and biomarker dynamics according to susceptible antibiotics must be revealed (19, 20).

To identify the longitudinal changes in hemodynamics and biomarkers according to antibiotic susceptibility in Gram-negative sepsis, we utilized the preclinical porcine bacteremia model induced with intravenous administration of ESBL-producing E. coli. Then, the porcine bacteremia model was allocated to two treatment groups, ertapenem and ceftriaxone, which are susceptible and insusceptible antibiotics to ESBL-producing E. coli, respectively.

## RESULTS

**FIG 1:**
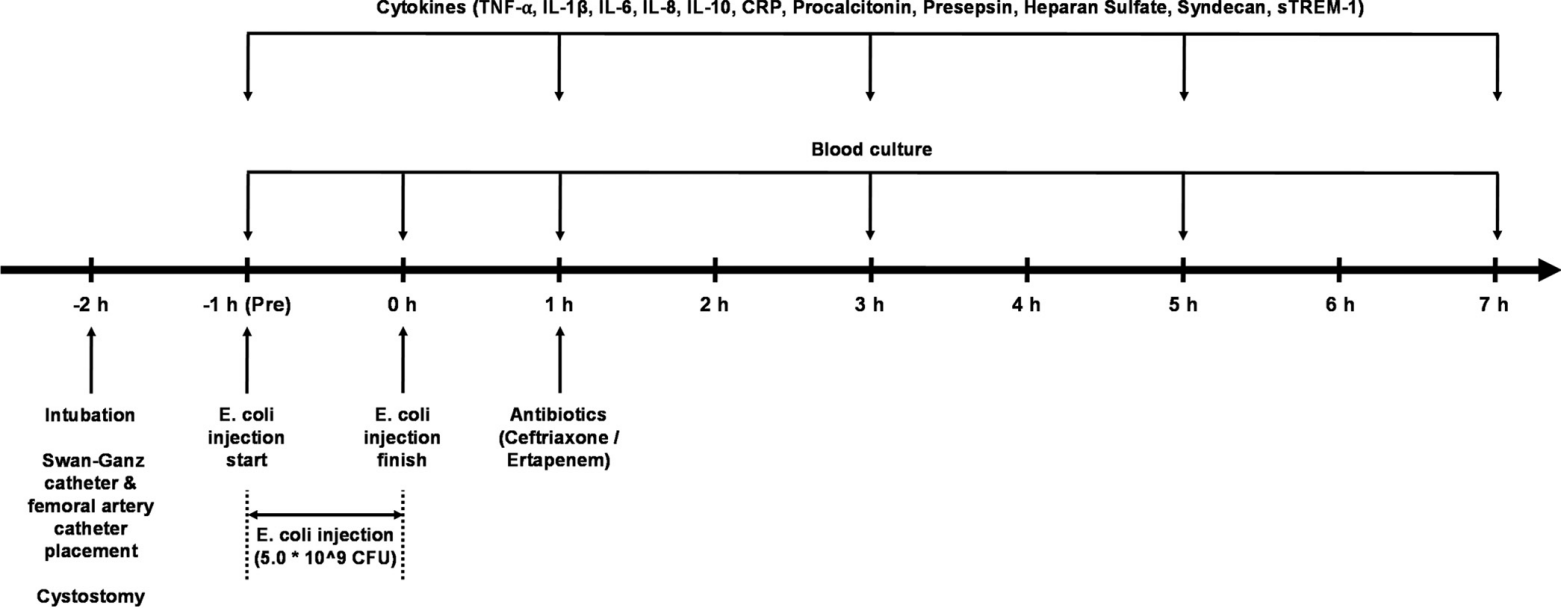
Schematics of animal experiments.

**FIG 2:**
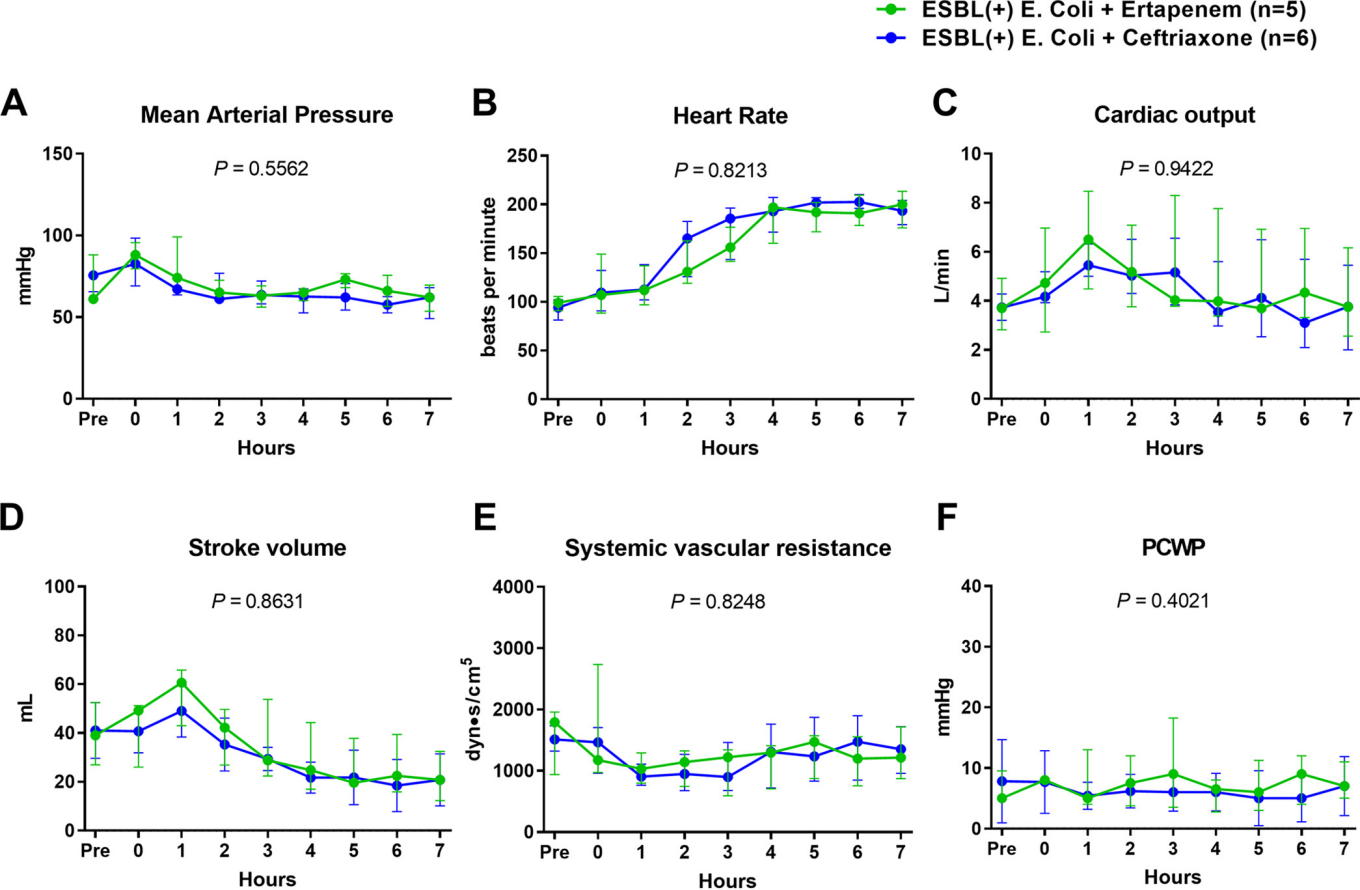
Comparisons of trends of hemodynamic variables between the ertapenem and ceftriaxone groups in the ESBL-producing E. coli-induced porcine bacteremia model. Data are presented as the medians and interquartile ranges. *P* values of times × groups interaction are denoted in each graph (two-way RM ANOVA). ESBL: extended-spectrum beta lactamases; MAP: mean arterial pressure; HR: heart rate; PCWP: pulmonary capillary wedge pressure.

**FIG 3:**
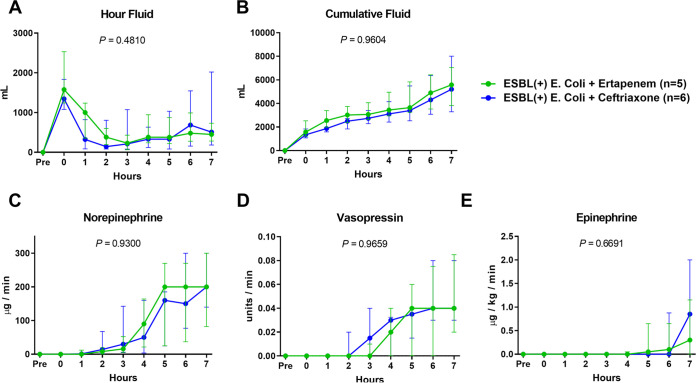
Comparisons of trends of administered fluid and vasopressors between ertapenem and ceftriaxone groups in an ESBL-producing E. coli-induced porcine bacteremia model. Data are presented as the medians and interquartile ranges. *P* values of times × groups interaction are denoted in each graph (two-way repeated measures analysis of variance).

**FIG 4:**
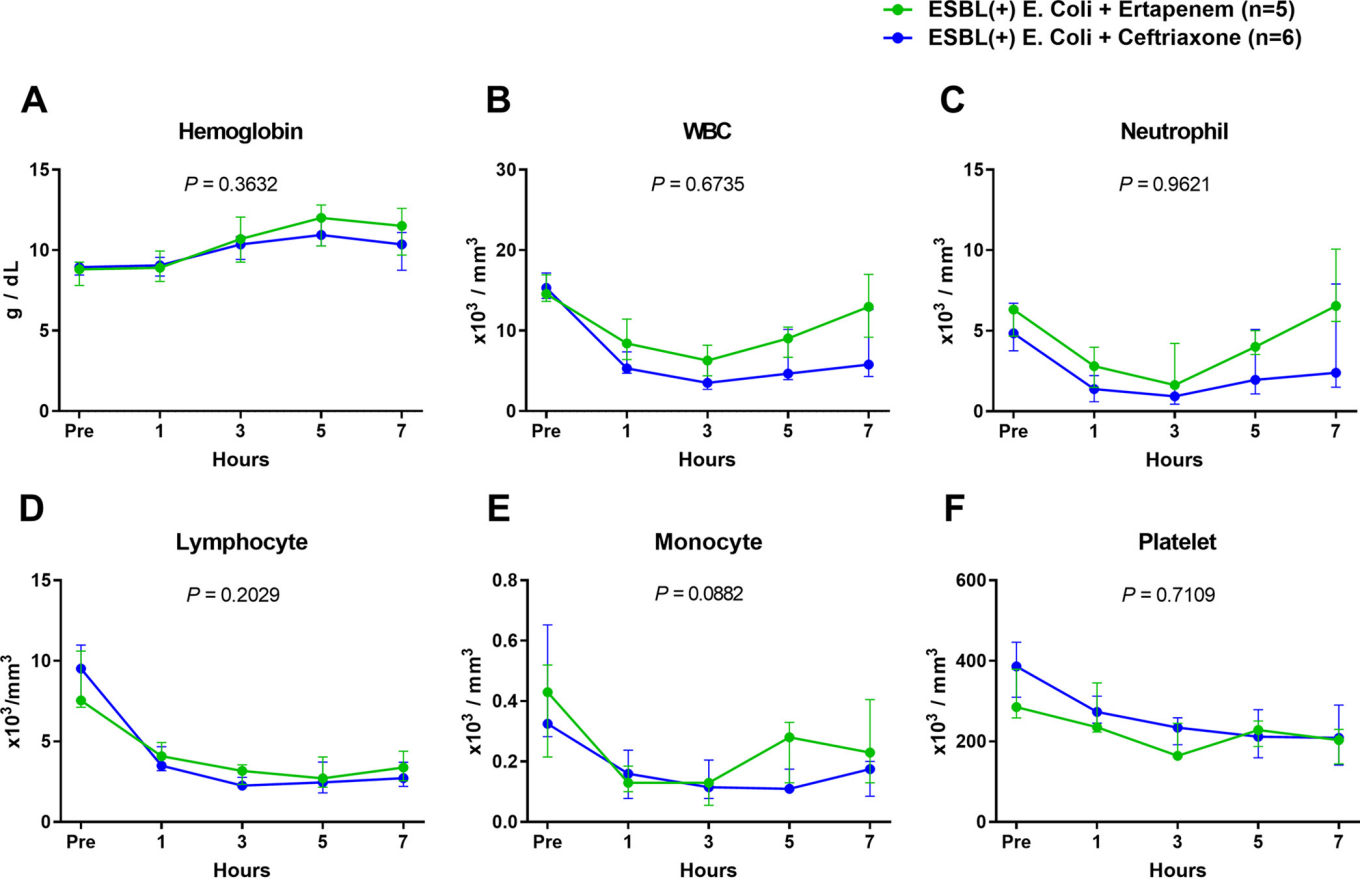
Comparisons of trends of blood cell counts between the ertapenem and ceftriaxone groups in the ESBL-producing E. coli-induced porcine bacteremia model. Data are presented as the medians and interquartile ranges. *P* values of times × groups interaction are denoted in each graph (two-way RM ANOVA). WBC: white blood cell; Hb: hemoglobin.

The pigs were monitored up to 7 h after the administration of ESBL-producing E. coli (Fig. 1). Five animals with ertapenem and six animals with ceftriaxone completed the study. The two groups showed no significant difference in hemodynamic variables, including mean arterial pressure (MAP) (Fig. 2A), heart rate (HR) (Fig. 2B), cardiac output (Fig. 2C), stroke volume (Fig. 2D), systemic vascular resistance (Fig. 2E), and pulmonary capillary wedge pressure (PCWP) (Fig. 2F). The amount of fluid (Fig. 3A and B) and dosage of the vasopressor (Fig. 3C–E) that was needed to maintain the mean arterial pressure above 65 mmHg were not significantly different between the two groups over time. Hemoglobin (Fig. 4A) and total white blood cell count (Fig. 4B) including the composite neutrophils (Fig. 4C), lymphocytes (Fig. 4D), and monocytes (Fig. 4E) were not significantly different between the two groups over time. Platelets in both groups decreased as a result of the progression of sepsis, without a significant difference between the two groups over time (Fig. 4F).

**FIG 5:**
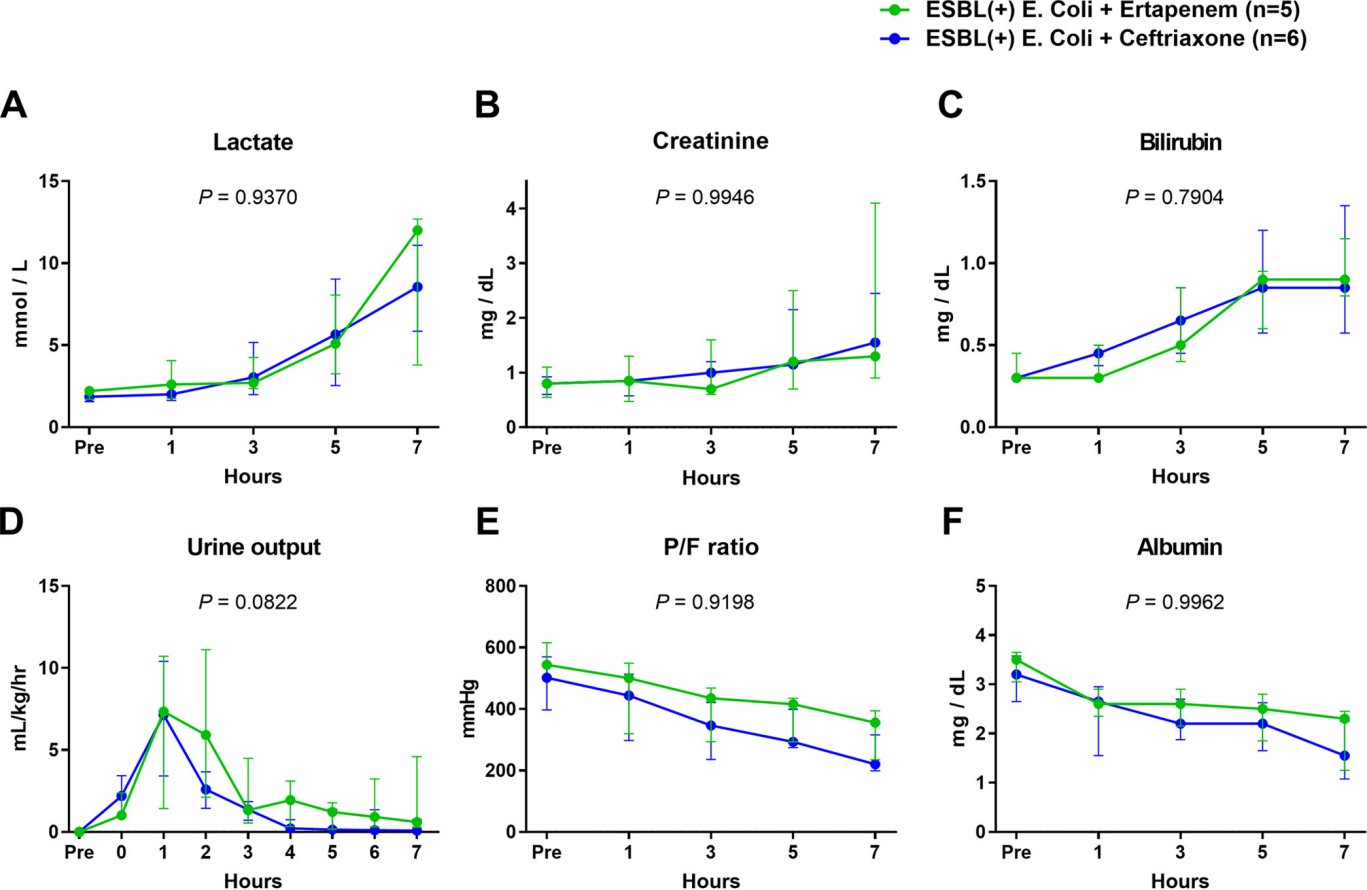
Comparisons of trends of lactate, creatinine, bilirubin, urine output, P/F ratio, and albumin between the ertapenem and ceftriaxone groups in ESBL-producing E. coli-induced porcine bacteremia model. Data are presented as the medians and interquartile ranges. *P* values of times × groups interaction are denoted in each graph (two-way RM ANOVA). P/F ratio: The ratio of arterial oxygen (PaO_2_) to fraction of inspired oxygen (FiO_2_).

To assess organ failure in this model, relevant values were measured (Fig. 5). As the bacterial injection progressed to sepsis, lactate (Fig. 5A), creatinine (Fig. 5B), and bilirubin (Fig. 5C) increased, while urine output (Fig. 5D), the ratio of arterial oxygen (PaO_2_) to fraction of inspired oxygen (FiO_2_) (P/F) ratio (Fig. 5E), and albumin (Fig. 5F) decreased. There were no significant differences in lactate, creatinine, bilirubin, urine output, P/F ratio, and albumin between two groups. The P/F ratio was found to be lower in the ceftriaxone group than in the ertapenem group, but statistical significance was not attained (*P < *0.0001 between times, *P = *0.2810 between groups, *P = *0.9198 between times × groups).

Based on the hemodynamics and laboratory data, the sequential organ failure assessment score (SOFA) score without the central nervous system (CNS) was evaluated to reflect the objective response (Fig. S1 in the supplemental material) (21). The total SOFA score was increased after bacterial administration, and the total SOFA score in the ceftriaxone group tended to be higher than that in the ertapenem group after 2 h due to the respiratory SOFA score, but without statistical significance. The trend of the total SOFA score as well as the SOFA score of each system was not significantly different between the two groups (*P < *0.0001 between times, *P = *0.4674 between groups, *P = *0.7086 between times × groups).

Before the E. coli injection (Pre), no bacteria were identified in the blood culture test (Fig. S2 in the supplemental material). E. coli were identified in all specimens at the time of completion of E. coli administration (0 h) and up to 1 h after administration. In the ertapenem group, all of the blood culture tests after 2 h of E. coli administration, which corresponds to 1 h after antibiotic treatment, presented negative results while the ceftriaxone group presented positive results.

**FIG 6:**
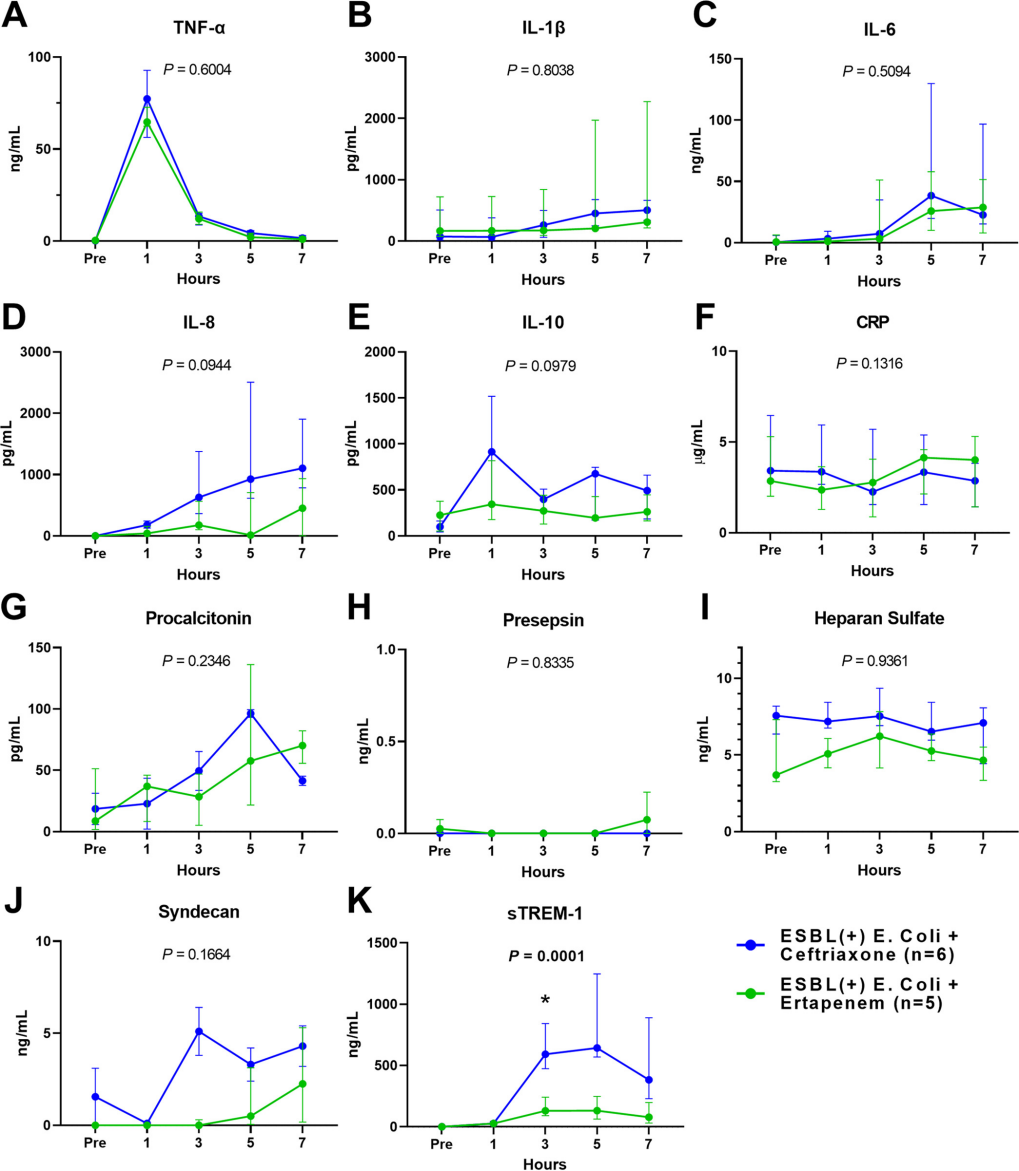
Comparisons of cytokine trends (TNF-α, IL-1β, IL-6, IL-8, IL-10, CRP, procalcitonin, presepsin, heparan sulfate, syndecan, and sTREM-1) between the ertapenem and ceftriaxone groups in the ESBL-producing E. coli-induced porcine bacteremia model. Data are presented as the medians and interquartile ranges. * denotes a significant difference between variables (*P = *0.0239, Sidak’s multiple comparisons). *P* values of times × groups interaction are denoted in each graph (two-way RM ANOVA).

Biomarkers were measured 1, 3, 5, and 7 h before the completion of E. coli injection (Fig. 6). Tumor necrosis factor (TNF)-α (Fig. 6A) peaked at 1 h after administration, followed by a decrease, while interleukin (IL)-1β (Fig. 6B), IL-6 (Fig. 6C), and IL-8 (Fig. 6D) increased during the observation period. Nevertheless, no significant difference in the trends of inflammatory cytokines, including TNF-α, IL-1β, IL-6, IL-8, and IL-10 (Fig. 6E), between the two groups was identified. Trends in clinical biomarkers such as C-reactive protein (CRP) (Fig. 6F), procalcitonin (Fig. 6G), and presepsin (Fig. 6H) were not significantly different between the two groups. Trends of heparan sulfate (Fig. 6I) and syndecan (Fig. 6J), which constitute the endothelial glycocalyx layer, also were not significantly different between the two groups. Soluble triggering receptor expressed on myeloid cells-1 (sTREM-1) (Fig. 6K) showed an increase from 1 h after bacterial injection, and the trends of sTREM-1 were significantly different between the two groups (*P < *0.0001 between times, *P = *0.0036 between groups, *P = *0.0001 between times × groups); a significant difference was observed 3 h after bacterial administration (2 h after antibiotics) (*P = *0.0239, Sidak’s multiple comparisons).

## DISCUSSION

In this study, using the E. coli-induced bacteremia porcine model, we demonstrated trends in hemodynamics and biomarkers during the progression of sepsis, including the difference in trends between the susceptible and insusceptible antibiotic groups. Among the hemodynamics and biomarkers, we identified that trends of sTREM-1 were significantly different between the two groups, which resulted in a significantly different level at post-antibiotic treatment periods.

TNF-α, a well-known proinflammatory biomarker, was the most sensitive biomarker at 1 h after the intravenous injection of E. coli but then decreased to baseline levels 7 h after the administration period, which was consistent with results of a previous study (22–24). The dynamics of TNF-α suggest that it may be valuable as a sensitive biomarker in bloodstream infection. However, TNF-α has limitations due to the short window period for detection (25). Furthermore, because no significant differences were identified in the post-antibiotic period between the two groups, TNF-α might have little discriminative power between the two groups, suggesting a limited perspective as a biomarker for *in vivo* antibiotic susceptibility. IL-1β, IL-6, and IL-8 continuously increased, and IL-10 was maintained or decreased after bacterial induction, which is consistent with the results of previous studies (22, 26). However, all interleukins were found to show no difference in trends between susceptible and insusceptible antibiotics. The trends in widely used clinical biomarkers such as CRP, procalcitonin, and presepsin also were not significant between the two groups (27). This might be derived from the delayed kinetic characteristics of biomarkers for the time to detectable increase and peak (12). Moreover, presepsin, which was presumed to peak at 3 h, was undetectable in every period, and CRP and procalcitonin exhibited differences even in the baseline period, suggesting either the limited reliability of the ELISA kit or individual differences of study subjects (28).

sTREM-1, a soluble form of triggering receptor expressed on myeloid cells, stands as an amplifier of the innate immune response in which the membrane-bound form (TREM-1) is expressed on neutrophils, mature monocytes and macrophages (29, 30). Lipopolysaccharide stimulation induced upregulation of TREM-1 in monocytes and neutrophils, followed by release of the soluble form of TREM-1 in blood via cleavage of the membrane-bound receptor (31). In a recent study, TREM-1 activation generated neutrophil extracellular trap (NET) structures, and inhibition of TREM-1 decreased NET release with a subsequent reduction in endothelial cell activation and vascular dysfunction (32). Based on our finding and the short half-life of sTREM-1(12), susceptible antibiotic treatment in bacteremia may prevent further release of sTREM-1, resulting in lower levels of sTREM-1 post-antibiotic treatment. However, the sTREM-1 level in the insusceptible group also decreased at 7 h, which leaves limited availability of a short window period for detecting sTREM-1 levels in the post-antibiotic period. For identification of the *in vivo* susceptibility to antibiotics, our data suggest that blood sampling after antibiotic administration may be effective at 2 h post-antibiotic administration.

In previous studies, the differential value of sTREM-1 was evaluated in the diagnosis of sepsis in populations of systemic inflammatory response syndrome and outperformed CRP and procalcitonin (33, 34). Moreover, a recent study of 293 patients with septic shock assessed that sustained elevation of sTREM-1 levels may be associated with a poor outcome (35). Despite its outstanding performance as a biomarker for the diagnosis and prognosis of sepsis, the trend of sTREM-1 as a dynamic biomarker of *in vivo* antibiotic susceptibility in sepsis has not been previously evaluated, highlighting its novel role in this study. Further clinical studies addressing the association of trends of sTREM-1 levels and antibiotic susceptibility are needed.

Long-term observation, including the prognosis of experimental subjects, was unfeasible due to the limitation of the study design. Instead, our study demonstrated negative conversion of blood cultures, which could be interpreted as a decrease in pathogen load to clinically undetectable levels as a consequence of effective antibiotics. In our previous pilot experiments, the median survival of pigs (*n* = 10) with ESBL-producing E. coli (5.0 * 10^9 CFU) administration was 10 h, complicating the acquisition of biomarkers over long periods of time.

This study has several limitations. First, the study was performed in a preclinical animal model, and differences in biomarkers may exist between humans and porcine models. Furthermore, due to the scarcity of biomarker data and antibodies in swine models, the efficacy and reliability of detection antibodies in several biomarkers (CRP, procalcitonin, presepsin, and glycocalyx composites) may be limited. In fact, the level of sTREM-1 after the bacterial injection in this study was higher than the usual level in patients with sepsis (33, 34). This might have derived from the difference in species or the modeling method of bacteremia (direct intravenous injection of a large amount of bacteria). Second, the control group was not included to evaluate the difference between the healthy population and bacteremia subjects. However, the baseline (pre) value may represent the variables of the control group. Third, although the porcine model was configured to represent patients with septic shock, initial hyperdynamic status after intravenous injection was rapidly followed by hypodynamic features that may limit our findings. Fourth, subject variations of the host and its immune system may exist. To maximally minimize individual bias, we brought pigs from a single farm and bred them in equal conditions. Fifth, as a single type of Gram-negative bacteria (E. coli) was utilized in this study, it cannot be extrapolated to other organisms, particularly Gram-positive bacteria (methicillin-resistant Staphylococcus aureus), which do not contain lipopolysaccharides. Although larger sample sizes and the use of multiple antibiotics against multiple bacteria species could broaden the scope of this study, practical limitations with the porcine model exist. However, although it may vary from region to region, we have selected the most common pathogens with significant issues with resistance to prescribed antibiotics, which are of widespread interest (36, 37). Furthermore, a clinical study that supplements our findings is planning to be executed to address the issue. Finally, our intravenous bacterial injection model was designed to be as reliable as possible, which may not represent other sources of infectious models such as pulmonary, genitourinary, or abdominal infections.

### Conclusion.

We implemented the E. coli-induced porcine sepsis model and identified the trends of hemodynamics and biomarkers with susceptible antibiotics compared to those of the insusceptible group. Among hemodynamic variables and biomarkers, the trend of sTREM-1 showed a significant difference with the use of susceptible antibiotics, which suggests sTREM-1 as a biomarker candidate of *in vivo* antibiotic susceptibility in Gram-negative sepsis. Further clinical study is warranted for the validation of sTREM-1 as a biomarker of *in vivo* antibiotic susceptibility in human bacteremia.

## MATERIALS AND METHODS

### Study settings.

The experiment was approved by the Institutional Animal Care and Use Committee of Seoul National University Bundang Hospital (protocol No. BA-2104-317-029-03), and all animals received care according to the Guide for the Care and Use of Laboratory Animals of the National Institutes of Health.

### Animal preparation.

Eleven healthy male three-way crossbred (Yorkshire × Berkshire × Duroc, Cronex, Seoul, Rep. of Korea) domestic pigs (44 ± 3.5 kg) were utilized in this study. Before the experiment, subjects were accommodated in an animal facility with standardized access to food and water. Anesthesia was induced via an intramuscular administration of a mixture of zolazepam/tiletamine (Zoletil, 5 mg/kg; Virbac, Carros, France) and xylazine (Rompun, 5 mg/kg; Elanco, Greenfield, IN). After the pigs were scrubbed with povidone-iodine soap, the bilateral neck and inguinal areas were shaved. The pigs were monitored with a 3-lead electrocardiography monitor, a pulse oximeter, and a rectal temperature probe (IntelliVue, Patient Monitor MP20; Philips, Amsterdam, Netherlands). Intubation was performed with a 7-Fr endotracheal tube that was applied to a mechanical ventilator (Drager Fabius GS, Lubeck, Germany) with an adequate concentration (2~3%) of inhalation anesthesia (Sevoflurane; Baxter Inc., Deerfield, IL). Ventilation was set in the volume-controlled mode with a tidal volume of 6 mL/kg, fraction of inspired oxygen (0.21), positive end-expiratory pressure of 5 cmH_2_O, and respiratory rate of 12 − 15/min. Further mechanical ventilation was adjusted to maintain arterial PaO_2_ above 65 mmHg and arterial PaCO_2_ between 40 and 45 mmHg. Meticulous preoperative povidone-iodine dressing on the region of intervention was conducted, followed by the application of a sterile surgical drape on the abdomen. Using ultrasonography, a 6-Fr arterial catheter (Merit Medical, South Jordan, UT) was inserted into both femoral arteries for invasive blood pressure monitoring and repetitive blood sampling. A Swan-Ganz catheter (Model 131HF, 7 Fr; Edwards Lifesciences, Irvine, CA) was introduced into the right jugular vein to monitor cardiac output and PCWP. An additional central venous catheter (ARROW CVC; Teleflex, Morrisville, NC) was applied to the left jugular vein for intravenous administration of fluid and vasopressors. Suprapubic cystostomy was conducted, and a Foley catheter was placed to monitor the urine output. After the above aseptic procedure, blood samples, including cultures, were obtained from the arterial catheter (38).

### Induction of the porcine bacteremia model.

To induce a porcine bacteremia model, extended-spectrum β-lactamase (ESBL)-producing Escherichia coli (5.0 * 10^9 CFU, Strain: BAA-196, ATCC, Manassas, VA) was diluted in 1000 mL of normal saline and intravenously administered over the course of an hour to subjects (Fig. 1). After complete infusion of bacteria, the pigs were assigned to two groups of antibiotics, ceftriaxone (2 g, Cerixone, Chong Kun Dang, Seoul, Rep. of Korea) or ertapenem (2 g, Invanz, MSD, Whitehouse Station, NJ). One hour after the completion of bacterial infusion and acquisition of blood samples, including culture, antibiotics were administered to the subject as allocated.

The animals were monitored up to 7 h after completion of bacterial infusion, and the primary goal was to maintain over 65 mmHg of MAP. Balanced crystalloid solution (Plasma Solution A, HK inno. N, Seoul, Republic of Korea) was challenged when MAP was less than 65 mmHg, and vasopressors consisting of norepinephrine, vasopressin, and epinephrine were sequentially initiated when the cumulative fluid exceeded 30 mL/kg or target MAP was not achieved with bolus fluid.

### Measurements and calculations.

The following hemodynamic variables were acquired continuously and recorded every hour: arterial blood pressure, HR, lead II electrocardiographic variables, pulse oximetry variables, pulmonary arterial pressure, and central venous pressure. Measurement of cardiac output using the thermodilution technique was conducted every hour, and the median value is presented. Arterial blood gas analysis, including the P/F ratio (ratio of partial pressure of oxygen to the fraction of inspired oxygen), electrolyte and lactate variables (Nova CCX; Nova, Waltham, MA), complete blood cell count (Hemavet 950FS; Drew Scientific Inc, Miami Lakes, FL), and chemistry panel test (albumin, creatinine, and bilirubin; VetScan; Abaxis, Union City, CA), were performed using blood samples obtained through the femoral arterial catheter at baseline and 1, 3, 5, and 7 h. The SOFA was calculated based on hemodynamic and laboratory variables (39). Blood samples (20 mL) for culture were obtained at baseline and 0, 1, 3, 5, and 7 h and injected in pairs of aerobic and anaerobic blood culture bottles (BD Bactec; Becton, Dickinson, Franklin Lakes, NJ). Then, the pairs of blood culture bottles were transferred without delay to the blood culture system (BD Bactec FX; Becton, Dickinson, Franklin Lakes, NJ) in a microbiologic laboratory. The final blood culture report was acquired from the microbiologic laboratory of the Department of Laboratory Medicine. Additional blood samples (10 mL) were collected in EDTA tubes (BD Vacutainer Systems; Becton, Dickinson, Franklin Lakes, NJ) at baseline and 1, 3, 5, and 7 h for cytokine analysis. The collected blood samples were centrifuged for 15 min at 3000 rpm, and the upper layer of plasma was aliquoted and stored at −70°C in a deep freezer until analysis. The levels of cytokines (TNF-α, IL-1β, IL-6, IL-8, and IL-10), CRP, procalcitonin, presepsin, heparan sulfate, syndecan, and sTREM-1 were measured using an enzyme-linked immunosorbent assay (ELISA) kit (DY690B, DY686, DY681, DY535, DY2648, P1000, Quantikine; R&D Systems Inc., Minneapolis, MN; MBS705285, MBS265068, MBS2018901, MBS923990, MBS2505397; MyBioSource Inc., San Diego, CA) based on the manufacturer’s instructions.

### Statistical analysis.

Variables are described as medians and interquartile ranges. Two-way analysis of variance for repeated measures with Sidak’s multiple-comparison test (two-way RM ANOVA) was performed to compare trends of variables between the two groups. *P* values less than 0.05 were considered significant. Statistical analyses and graph generation were performed using Prism 9.2 (GraphPad Software Inc., San Diego, CA).
