# Supplementary material for: Changes in Biomarkers and Hemodynamics According to Antibiotic Susceptibility in a Model of Bacteremia
Source: Microbiol Spectr. 2022 Jul 11;10(4):e00864-22. doi: 10.1128/spectrum.00864-22 (PMC9430499; doi:10.1128/spectrum.00864-22)
Supplement: Supplemental file 1 — Supplemental material. Download spectrum.00864-22-s0001.pdf, PDF file, 0.4 MB [file spectrum.00864-22-s0001.pdf]

Online Data Supplement for

Changes in biomarkers and hemodynamics according to antibiotic susceptibility in a model of bacteremia

Inwon Park, Dongsung Kim, Jae Hyuk Lee*, Hwain Jeong, Sumin Baek, Seonghye Kim, Serin Kim, Ji Eun Hwang, Hyuksool Kwon, You Hwan Jo

* Corresponding authors. E-mail: hyukmd@gmail.com (J.H.L)

This material includes:

Supplementary figure 1. Comparisons of trends of SOFA (without CNS) score between ertapenem and ceftriaxone group in ESBL-producing E. coli-induced porcine bacteremia model.

Supplementary figure 2. Blood culture reports of ertapenem and ceftriaxone group in ESBL-producing E. coli-induced porcine bacteremia model.

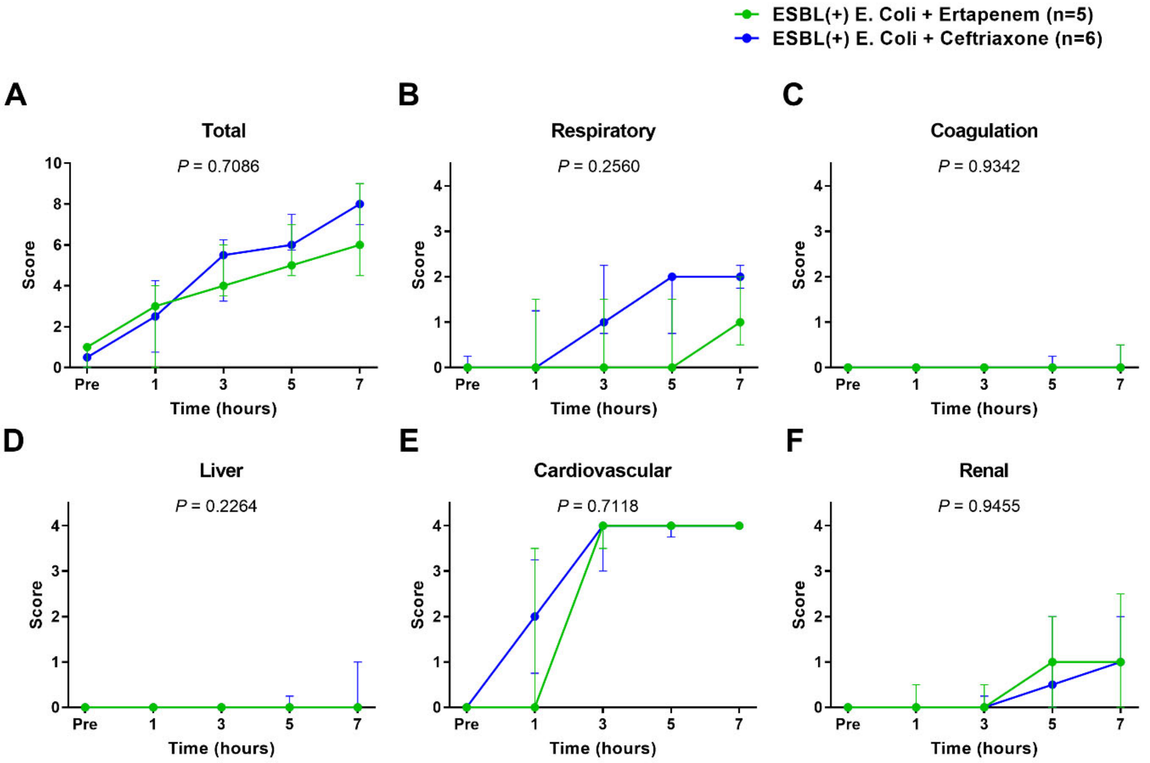

Supplementary figure 1. Comparisons of trends of SOFA (without CNS) score between ertapenem and ceftriaxone group in ESBL-producing E. coli-induced porcine bacteremia model. Data are presented as the median and interquartile range. P values of groups * times interaction are denoted in each graph (Two-way RM ANOVA).

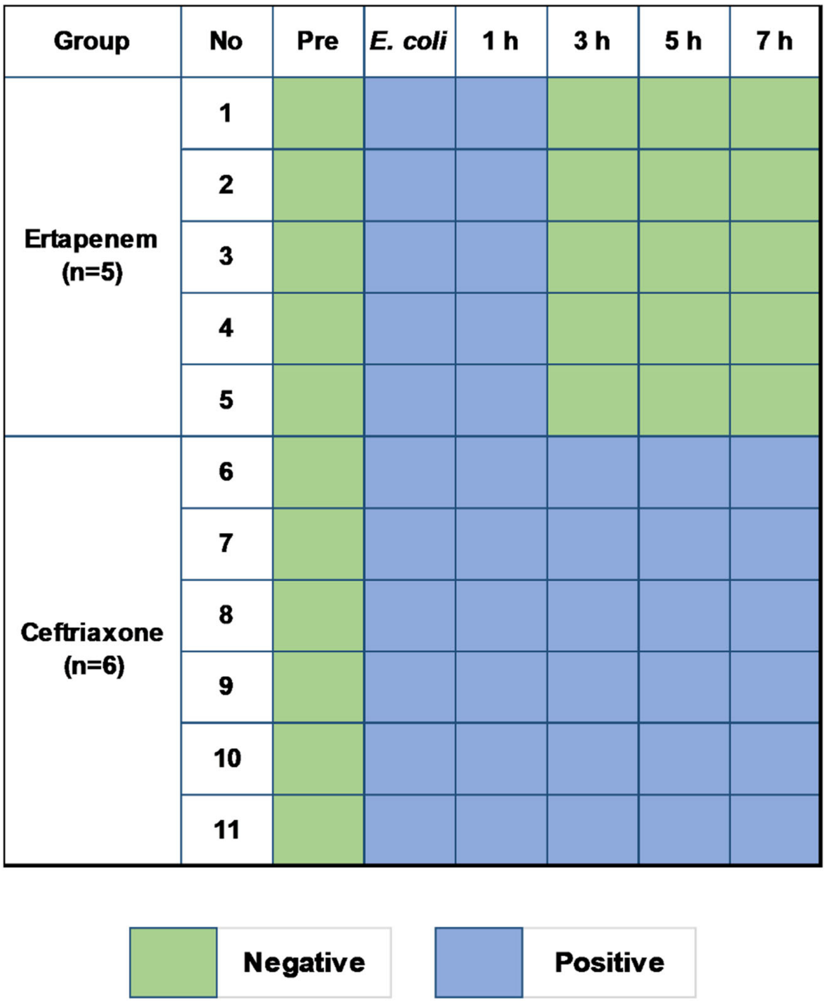

Supplementary figure 2. Blood culture reports of ertapenem and ceftriaxone group in ESBL-producing E. coli-induced porcine bacteremia model.
